# Sleep and APOE‐ε4 have a synergistic effect on plasma biomarkers and longitudinal cognitive decline in older adults

**DOI:** 10.1111/cns.14558

**Published:** 2024-02-07

**Authors:** Xianfeng Yu, Xia Zhou, Zhengbo He, Beiqi He, Ke Wan, Min Wei, Tengfei Guo, Ying Han

**Affiliations:** ^1^ Department of Neurology Xuanwu Hospital of Capital Medical University Beijing China; ^2^ Department of Neurology The First Affiliated Hospital of Anhui Medical University Hefei China; ^3^ Institute of Biomedical Engineering Shenzhen Bay Laboratory Shenzhen China; ^4^ School of Information and Communication Engineering Hainan University Haikou China; ^5^ Center of Alzheimer's Disease Beijing Institute for Brain Disorders Beijing China; ^6^ National Clinical Research Center for Geriatric Disorders Beijing China

**Keywords:** Alzheimer's disease, APOE ε4, biomarkers, discrimination, sleep

## Abstract

**Background:**

Sleep disorders are prevalent among patients with Alzheimer's disease (AD), and the APOE ε4 genotype is a key genetic risk factor for sporadic AD. However, the combined effect of the genotype and sleep disorders on cognitive decline remains uncertain.

**Methods:**

A total of 972 participants were drawn from the SILCODE cohort, comprising 655 without the ε4 allele (APOE−) and 317 with ε4 allele (APOE+). Data were collected, including neuropsychological assessments, sleep measurements, plasma biomarkers, and PET imaging. A Sleep Composite Index (SCI) was created, categorizing participants into high risk (Sleep+) and low risk (Sleep−).

**Results:**

Significant predictions of dementia risk associated with plasma p‐tau181, neurofilament light chain (NfL), and SCI. Individuals with both Sleep+ and APOE+ had a higher risk of dementia compared to those with Sleep‐. The Sleep+/APOE+ group had higher plasma NfL levels than the Sleep−/APOE− group. Similar trends emerged in plasma NfL levels among the Aβ PET‐positive subgroup. Plasma NfL levels explained 23% of the relationship between SCI and cognitive impairment.

**Conclusion:**

Our study highlights sleep disorder was associated with cognitive decline, with plasma NfL playing a partial mediating role. These findings explain how sleep disorders affect cognitive function and emphasize the importance of healthy sleep for older adults.

## Graphical abstract

Our study highlights that sleep disorder was associated with cognitive decline, with plasma NfL playing a partial mediating role. These findings explain how sleep disorders affect cognitive function and emphasize the importance of healthy sleep for older adults.

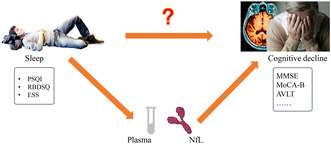

## 1 INTRODUCTION

Alzheimer's disease (AD) is a neurodegeneration disease characterized by its notable detrimental impairment of memory loss and declines in other cognitive functions.^1^ While patients with AD experience dysfunctions caused by neurodegeneration and cognitive impairments, one of the other associated disruptions is sleep disorder. Sleep disorder and related disruptions are common in patients with AD, with nearly 60% of individuals reporting sleep disorder.^2^ Individuals with sleep disorders often experience an alteration in sleep architecture and sleep–wake cycle, resulting in increased daytime sleepiness, sundowning (emotional impulsiveness in the nighttime), and insomnia.^3^ Mild cognitive impairment (MCI) is considered the first clinical stage of AD, and patients with MCI also exhibit more sleep disruption than the normal population.^4^ Additionally, it has been reported that these sleep disorders may be related to the pathophysiological mechanism of neurodegeneration in MCI patients.^5^

Sleep disorder is not solely the accompanying symptoms of AD progression; it contributes to cognitive decline and AD progression.^6^ A bidirectional hypothesis between sleep disorder and AD progression was recently proposed, in which sleep disorder mutually interacts with AD‐related pathophysiological biomarkers, such as amyloid‐β (Aβ) and hyperphosphorylated tau protein (p‐tau).^7^, ^8^ Aβ peptide and hyperphosphorylated tau protein are two dominant hallmarks of AD, and are associated with impairments in memory and executive function.^9^ Some studies revealed the potential utilization of neurofilament light chain (NfL) and glial fibrillary acidic protein (GFAP) concentration levels as biomarkers of sleep disorder and daytime drowsiness, as well as their roles in predicting decline in neurodegeneration and cognitive performance.^10^, ^11^

Besides, apolipoprotein E gene ε4 allele (APOE ε4) is one of three variants of the APOE gene, which locus on chromosome 19, together with other alleles of ε2 (APOE ε2) and ε3 (APOE ε3).^12^ While APOE ε2 is found to be protective in reducing risk of AD, APOE ε4 is associated with AD pathophysiological biomarker progression. Compared with non‐APOR ε4 carriers, APOE ε4 carriers show increases in cerebrospinal fluid (CSF) p‐tau levels as early as about 50 years.^13^ Besides, APOE ε4 carriers have higher Aβ plague burden and more severe neurofibrillary tau tangles than APOE ε4 non‐carriers, which indicates higher risks of progressing to dementia.^14^

However, the synergistic effect between sleep disorder and APOE ε4 genotype on cognitive decline remains unclear. An exploratory attempt using a mouse model demonstrated a positive‐feedback loop that APOE ε4‐encoding mice have accelerated increases in Aβ plague and reduced microglia around Aβ‐plague, which in turn results in further sleep deprivation.^15^ Nevertheless, very few studies attempted to investigate their synergy effect in humans, so it is necessary to have a deeper and more comprehensive look into whether their interactions increase risk conversion to dementia.

In the present study, we analyzed the participants from the Sino Longitudinal Study on Cognitive Decline (SILCODE) study, an ongoing prospective cohort study centered on Xuanwu Hospital in Beijing,^16^ to derive a sleep composite measurement composed of nighttime sleep quality, rapid eye movement sleep behavior disorder, and sleepiness in daily activities. The aim of this study is to investigate whether APOE ε4 allele exacerbates the impact of sleep disorder on cognitive decline over time, which may help us understand more about how APOE ε4 and sleep disorder relate to cognitive decline in elderly adults.

## 2 METHODS

### 2.1 Participants

All participants were recruited from the SILCODE cohort, which centered on Xuanwu Hospital in cooperation with an alliance of 94 hospitals from 50 cities in China. Specific inclusion/exclusion criteria can be found at https://www.clinicaltrials.gov/ct2/show/study/NCT03370744. In our study, patients with MCI and AD were defined as individuals with cognitively impaired (CI). The definition of MCI was in accordance with the criteria proposed by Jak and Bondi in 2014,^17^ which met any one of the following three conditions and failed to meet the criteria for dementia: (1) having impaired scores (defined as >1 SD below the age/education‐corrected normative means) on both measures in at least one cognitive domain (memory, language, or speed/executive function); (2) having impaired scores in each of the three cognitive domains (memory, language, or speed/executive function); and (3) the Functional Activities Questionnaire (FAQ) ≥ 9. Patients with AD dementia were diagnosed according to the Diagnostic and Statistical Manual of Mental Disorders (fifth edition), and the guidelines for dementia due to AD were proposed by the National Institute on Aging and Alzheimer's Association (NIA‐AA) workgroups.^18^ Both baseline and the first follow‐up assessments were performed at Xuanwu Hospital, and the follow‐up interval for each patient was 1 year.

The exclusion criteria included (1) a history of stroke; (2) major depression, with Hamilton Depression Scale (HAMD) score > 24 points; (3) other central nervous system diseases that may cause cognitive impairment, such as Parkinson's disease, tumors, encephalitis, and epilepsy; (4) traumatic brain injury; (5) systemic diseases, such as thyroid dysfunction, syphilis, and acquired immunodeficiency syndrome (AIDS); and (6) psychosis or congenital mental developmental delay. Following the exclusion criteria, a total of 972 participants were included in the present study. The study was approved by the Ethics Committee of Xuanwu Hospital of Capital Medical University and conducted in accordance with ethical standards.

### 2.2 Measurements

#### 2.2.1 Neuropsychological assessments

Demographical and clinical data were collected, including age, gender, and years of education, as well as clinical data on history of hypertension, diabetes, hyperlipidemia, and coronary heart disease. General cognitive performance was measured by the Mini‐Mental State Examination (MMSE) and the Chinese version of the Montreal Basis for Cognitive Assessment (MoCA‐B).^19^ To measure impairment in episodic memory and executive function, data of other neuropsychological measurements were also collected: 30‐minute delayed free recall in Auditory Verbal Learning Test (AVLT‐N5) and AVLT recognition task (AVLT‐N7),^20^ sections A and B in the Shape Trail Test (STT‐A and STT‐B).^21^ Measurement of mood alteration, namely Geriatric Depression Scale (GDS), HAMD, and Hamilton Anxiety Scale (HAMA), was also included. The specialties of neurology and neuropsychology were involved in the adjudication of diagnoses and conversion determination and interpreted information from the neuropsychological testing data.

#### 2.2.2 Plasma biomarker measurement

The current study adopted the plasma biomarker measurement pipeline of the SILCODE study. In brief, the single‐molecule array (Simoa) p‐tau181 Advantage Kit was used to measure p‐tau181 concentration, and the Simoa Human Neurology 4‐Plex E (N4PE) assay (Quanterix) was used to measure Aβ40, Aβ42, NfL, and GFAP concentrations. All measurements for the five analytes were above the detection limit, and the intra‐assay variation coefficient was less than 10%. The data were then matched to phenotype information.

#### 2.2.3 Imaging acquisition protocol

The MRI and PET images were both acquired with a simultaneous hybrid PET/MR scanner (SIGNA; GE Healthcare, Chicago, IL, USA). Participants were instructed to keep their eyes closed and stay awake, to free themselves from thoughts, and to keep static as much as possible during imaging. Participants were given foam pads and headphones to cancel MR machine noise and minimize head movement.

T1‐weighted images were acquired with a magnetization‐prepared rapid gradient echo sequence: field of view (FOV) = 256 × 256 mm^2^, matrix = 256 × 256, slice thickness = 1 mm, gap = 0, slice number = 192, repetition time (TR) = 6.9 ms, echo time (TE) = 2.98 ms, inversion time = 450 ms, flip angle = 12°, and voxel size = 1 × 1 × 1 mm^3^. The images were acquired 50 min after intravenous injection with the tracer ^18^F‐Florbetapir of 7–10 mCi, and the data were recorded by using a time‐of‐flight ordered subset expectation maximization algorithm with the following parameters: scan duration = 20 min, 8 iterations, 32 subsets matrix = 192 × 192, FOV = 350 × 350, and half‐width height = 3.

#### 2.2.4 Sleep Composite Index (SCI)

To comprehensively measure individual's overall sleep quality from perspectives of nocturnal sleep quality and daytime drowsiness, we carefully selected three measurements that are clinically and academically widely used as components for SCI, namely Pittsburg Sleep Quality Index (PSQI),^22^ REM Sleep Behavior Disorder Screening Questionnaire (RBDSQ),^23^ and Epworth Sleepiness Scale (ESS),^24^ which, respectively, evaluate sleep quality, sleep disturbances, rapid eye movement (REM) sleep behavior disorder, and daytime drowsiness. A multivariate logistic regression analysis was applied to build the SCI as a composite indicator reflecting an individual's overall sleep situation. In detail, SCI was supposed to measure the quality of nighttime sleep, REM (sleep behavioral disruptions), and tendencies of daytime sleepiness. The formula for calculating the SCI is shown below: *Sleep Composite Index* = −3.914221 + PSQI*0.0555522 + RBDSQ*0.7287495 + ESS*0.0660809.

In the present study, participants were grouped based on their SCI score, participants who scored higher than the median score were grouped as high‐risk group (Sleep+), and those who scored below the median score were grouped as low‐risk group (Sleep−).

#### 2.2.5 APOE genotyping

DNA sequences for each subject were extracted for SNPs rs7412 and rs429358 from the APOE ε2/ε3/ε4 haplotype. APOE was genotyped using the standard Sanger sequencing method (Sangon, Shanghai, China) with the following primers: 5′‐ACGCGGGCACGGCTGTCCAAGG‐3′ (forward) and 5′‐GGCGCTCGCGGATGGCGCTGA‐3′ (reverse). APOE was amplified using the following conditions: 1 cycle of 98°C for 10 s, 35 cycles of 72°C for 5 s, and 1 cycle of 72°C for 5 min. PCR was performed in a final volume of 30 μL containing 10 pmol of forward and reverse primers, and 50 ng of genomic DNA template using PrimeSTAR HS DNA polymerase with the GC Buffer.^25^

### 2.3 Data analysis

#### 2.3.1 Neuroimaging data processing

First, all raw T1‐weighted and Aβ PET data were converted from DICOM to NIfTI file format (https://people.cas.sc.edu/rorden/mricron/dcm2nii.html). Second, PET images were coregistered on corresponding T1‐weighted images, followed by correction of partial volume effect based on Muller–Gartner algorithm.^26^ Third, T1‐weighted images were normalized to Montreal Neurological Institute (MNI) standard space using the Statistical Parametric Mapping (SPM8; https://www.fil.ion.ucl.ac.uk/spm/software/spm8), and corrected Aβ PET images were normalized to the MNI standard space using forward transformation referenced from T1‐weighted spatial normalization. Lastly, PET images were smoothed with an 8‐mm full‐width‐at‐half‐maximum Gaussian kernel. The current study used the whole cerebellum as the reference region, and the whole cerebral cortex was used as the region of interest. The average standard uptake value ratio (SUVR) was calculated with reference from the whole cerebral cortex, and cutoff SUVR value between Aβ negative and positive was set at 1.11.^27^

#### 2.3.2 Statistical analysis

The Kolmogorov–Smirnov test was used to determine whether the distribution of data was normal. Continuous variables were presented as the mean ± standard deviation (for normally distributed data) and as the frequency [percentage] (for categorical variables). Differences between groups were examined using Student's *t*‐test for continuous data, and Chi‐square test or Fisher's exact test for categorical data. The Kaplan–Meier method was used to build the survival curves to forecast the likelihood and timing of the conversion, and the log‐rank test (survminer R package) was used for further comparison. GraphPad Prism 9 and R 4.1.0 (https://www.r‐project.org/) were used to generate figures, and all statistical analyses were performed using R 4.1.0 with statistical significance defined as a two‐tailed *p*‐value <0.05.

## 3 RESULTS

### 3.1 Demographical and clinical characteristics

Demographical and clinical characteristics are summarized in Table 1. A total of 972 participants were recruited in the current study. Individual diagnosis, apolipoprotein E ε4 allele carriage, and sleep status at baseline are shown in Table 2. Among them, 314 participants underwent Aβ PET scanning (MCI: 44, 14.01%; AD: 28, 8.92%) and 361 participants received plasma measurement (MCI: 46, 12.74%; AD: 23, 6.37%). Mean age was 66.70 years (SD = 7.37) for APOE ε4 allele non‐carriage group (APOE−) and 67.55 years (SD = 7.26) for APOE ε4 allele carriage group (APOE+). 59.85% of participants in APOE‐ group and 70.66% in APOE+ group were female. APOE+ group exhibited significantly poor performance in both global cognition (MMSE, MoCA‐B) and memory function (AVLT‐N5 and N7), as well as executive function (STT‐A and STT‐B), compared to APOE− group. In terms of plasma markers, APOE− group versus APOE+ group showed Aβ42/40 (0.07 ± 0.05 vs. 0.08 ± 0.32), p‐tau181 (2.16 ± 1.08 vs. 3.46 ± 9.14, *p* < 0.05), NfL (17.43 ± 9.34 vs. 19.26 ± 14.71), and GFAP (121.39 ± 75.39 vs. 137.09 ± 75.36). The average total years of follow‐ups were 4.74 ± 2.85 for non‐carriers and 4.97 ± 2.81 for carriers. In the longitudinal study, the drop‐out rates of MCI and AD participants were 14.51% (141/972).

**TABLE 1:** Demographic, neuropsychological, and biomarker characteristics of the total sample and grouped by apolipoprotein E ε4 allele carriage.

Variable	Whole cohort	APOE− (*n* = 655)	APOE+ (*n* = 317)
Follow‐up time	4.73 ± 2.95	4.74 ± 2.85	4.97 ± 2.81
Age	67.14 ± 7.61	66.70 ± 7.37	67.55 ± 7.26
Female, % (*n*)	63.37 (616)	59.85 (392)	70.66 (224)
Education	12.05 ± 4.10	12.25 ± 3.72	12.15 ± 4.26
MMSE	26.96 ± 3.83	27.36 ± 3.48	26.32 ± 4.49*
HAMD	4.17 ± 4.23	4.12 ± 4.00	4.53 ± 4.50
HAMA	5.02 ± 4.60	4.72 ± 4.43	5.06 ± 4.42
AVLT‐N5	6.45 ± 2.90	6.77 ± 2.66	6.03 ± 3.28*
AVLT‐N7	21.49 ± 3.05	21.93 ± 2.46	20.75 ± 3.90*
STT‐A	73.14 ± 56.42	68.56 ± 46.27	81.94 ± 76.47*
STT‐B	161.16 ± 85.42	151.24 ± 69.05	173.47 ± 97.72*
VFT	18.72 ± 5.37	19.17 ± 5.02	17.66 ± 5.85*
BNT	25.06 ± 3.67	25.12 ± 3.46	24.69 ± 4.05
GDS	2.65 ± 2.46	2.48 ± 2.38	2.87 ± 2.48
MoCA‐B	24.78 ± 4.14	25.19 ± 3.57	24.06 ± 4.95*
SUVR (*n*)	1.04 ± 0.17 (314)	1.00 ± 0.12 (127)	1.12 ± 0.22 (187)*
PSQI	5.23 ± 4.82	5.05 ± 3.42	5.28 ± 3.53
RBDSQ	1.61 ± 3.94	1.65 ± 4.60	1.33 ± 1.79
ESS	6.82 ± 4.80	6.69 ± 4.63	6.93 ± 5.30
SCI	−4.27 ± 1.34	−4.27 ± 1.30	−4.14 ± 1.31
Plasma (*n*)	361	249	112
Aβ40	109.79 ± 94.44	106.31 ± 71.89	112.22 ± 119.79
Aβ42	6.14 ± 1.69	6.28 ± 1.54	5.78 ± 1.98*
Aβ42/40	0.07 ± 0.18	0.07 ± 0.05	0.08 ± 0.32
p‐tau181	2.55 ± 5.14	2.16 ± 1.08	3.46 ± 9.14*
NfL	17.91 ± 11.21	17.43 ± 9.34	19.26 ± 14.71
GFAP	125.90 ± 75.12	121.39 ± 75.39	137.09 ± 75.36

Abbreviations: APOE−, APOE ε4 allele non‐carriage; APOE+, APOE ε4 allele carriage; AVLT‐N5, Auditory Verbal Learning Test–Huashan version long‐delayed free recall (20 min); AVLT‐N7, AVLT–Huashan version long‐delayed recognition (20 min); BNT, Boston Naming Test; GDS, Geriatric Depression Scale; GFAP, glial fibrillary acidic protein; HAMA, Hamilton Anxiety Scale; HAMD, Hamilton Depression Scale; MMSE, Mini‐Mental State Examination test; MOCA‐B, Montreal Basis for Cognitive Assessment; NfL, neurofilament light chain; SCI, Sleep composite Index; STT‐A, Shape Trail Test A; STT‐B, Shape Trail Test B; VFT, Verbal Fluency Test (animal).

* *p* < 0.05 (APOE− vs. APOE+).

**TABLE 2:** Individual diagnosis and apolipoprotein E ε4 allele carriage and sleep status at baseline.

	NC	MCI	AD
ApoE+	199	73	45
ApoE−	523	92	40
Sleep+	127	148	68
Sleep−	296	27	25
Age	65.91 ± 6.65^a^ ^,^ ^b^	68.99 ± 8.71^c^	72.09 ± 9.38
Female, % (*n*)	63.85 (461)	60.00 (99)	64.71 (55)
Education	12.33 ± 3.80^b^	11.28 ± 4.28^c^	9.26 ± 5.05
MMSE	28.42 ± 1.99^a^ ^,^ ^b^	24.64 ± 3.88^c^	18.86 ± 4.76
HAMD	3.89 ± 4.00^a^	5.88 ± 5.54	4.72 ± 4.43
HAMA	4.54 ± 4.14^a^ ^,^ ^b^	5.97 ± 5.23^c^	7.38 ± 6.39
AVLT‐N5	7.31 ± 2.20^a^ ^,^ ^b^	3.24 ± 2.62^c^	0.59 ± 1.64
AVLT‐N7	22.31 ± 1.77^a^ ^,^ ^b^	19.20 ± 3.10^c^	13.21 ± 5.45
STT‐A	61.01 ± 21.85^a^ ^,^ ^b^	98.67 ± 47.54^c^	240.12 ± 168.55
STT‐B	140.88 ± 44.34^a^ ^,^ ^b^	234.80 ± 120.29^c^	345.76 ± 154.01
VFT	19.54 ± 4.74^a^ ^,^ ^b^	15.66 ± 4.91^c^	10.10 ± 6.11
BNT	25.53 ± 2.98^a^ ^,^ ^b^	23.05 ± 4.90^c^	18.97 ± 5.42
GDS	2.45 ± 2.31^a^ ^,^ ^b^	3.18 ± 3.00	3.81 ± 2.469
MoCA‐B	25.83 ± 2.74^a^ ^,^ ^b^	21.39 ± 4.77^c^	14.07 ± 5.27
SUVR (*n*)	0.99 ± 0.10 (251)^a^ ^,^ ^b^	1.13 ± 0.23 (39)^c^	1.32 ± 0.22 (24)
PSQI	5.18 ± 3.49^b^	5.37 ± 3.30^c^	3.14 ± 2.59
RBDSQ	1.57 ± 4.29^b^	1.93 ± 2.07^c^	0.74 ± 1.26
ESS	6.80 ± 4.67	6.87 ± 5.47	6.63 ± 6.04
SCI	−4.20 ± 1.30^a^ ^,^ ^b^	−4.60 ± 1.51^c^	−3.87 ± 0.94
Plasma (*n*)	290	48	23
Aβ40	109.60 ± 89.44^a^ ^,^ ^b^	96.722 ± 21.177	97.49 ± 16.04
Aβ42	6.17 ± 1.5	6.20 ± 2.18	5.10 ± 1.03
Aβ42/40	0.07 ± 0.04^b^	0.07 ± 0.15^c^	0.05 ± 0.01
p‐tau181	2.05 ± 0.96^a^ ^,^ ^b^	4.74 ± 13.62	4.04 ± 1.20
NfL	16.01 ± 7.26^a^ ^,^ ^b^	21.89 ± 15.72^c^	33.11 ± 22.84
GFAP	115.08 ± 58.23^a^ ^,^ ^b^	149.56 ± 111.40^c^	202.07 ± 110.39

Abbreviations: AVLT‐N5, Auditory Verbal Learning Test–Huashan version long‐delayed free recall (20 min); AVLT‐N7, AVLT–Huashan version long‐delayed recognition (20 min); BNT, Boston Naming Test; GDS, Geriatric Depression Scale; GFAP, glial fibrillary acidic protein; HAMA, Hamilton Anxiety Scale; HAMD, Hamilton Depression Scale; MMSE, Mini‐Mental State Examination test; MOCA‐B, Montreal Basis for Cognitive Assessment; NfL, neurofilament light chain; SCI, Sleep composite Index; STT‐A, Shape Trail Test A; STT‐B, Shape Trail Test B; VFT, Verbal Fluency Test (animal).

^a^ *p* < 0.05 (NC vs. MCI).

^b^ *p* < 0.05 (NC vs. AD).

^c^ *p* < 0.05 (MCI vs. AD).

### 3.2 The association of plasma biomarkers, sleep index, and longitudinal progression to dementia

**FIGURE 1:**
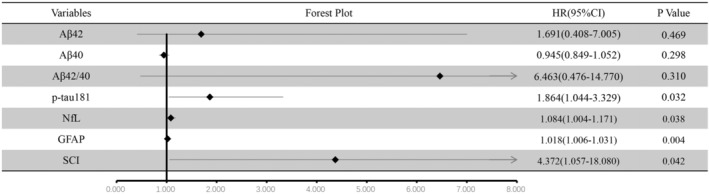
Estimation of various biomarkers on future conversion to dementia. GFAP, glial fibrillary acidic protein; HR, hazard ratio; NfL, neurofilament light chain; SCI, Sleep Composite Index.

Figure 1 shows the estimation of six plasma biomarkers and SCI on the future conversion to dementia. Plasma Aβ42, Aβ40, and Aβ42/40 did not exhibit significant effects on dementia conversion. However, plasma p‐tau181 (hazard ratio (HR): 1.864, 95% confidence interval (CI) 1.044–3.329), plasma NfL (HR: 1.084, 95% CI 1.004–1.171), and plasma GFAP (HR: 1.018, 95% CI 1.006–1.031) showed statistically significant associations with the risk of conversion (*p* < 0.05). Additionally, SCI also demonstrated a promising predictive role (HR: 4.372, 95% CI 1.057–18.080, *p* < 0.05) in the risk of conversion.

### 3.3 The association of SCI with plasma biomarkers

**FIGURE 2:**
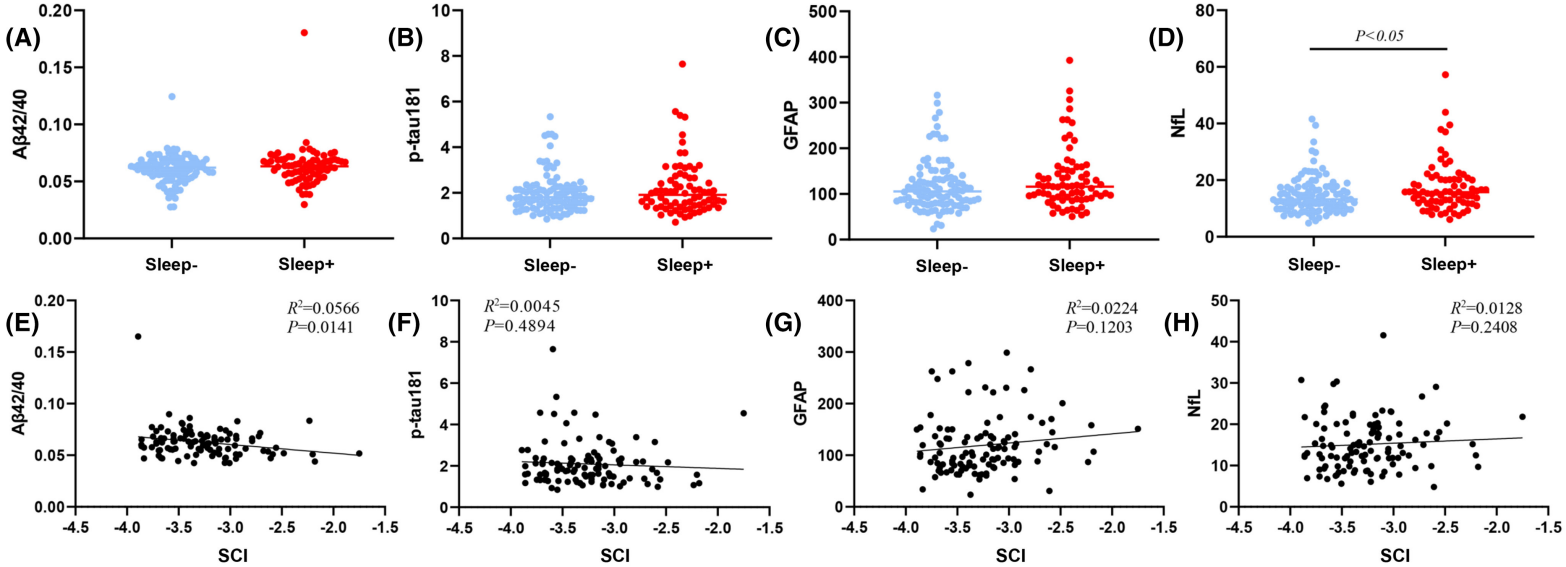
Relationship between SCI and plasma biomarkers. (A–D) Group differences in plasma biomarkers grouped by SCI score; and (E–H) relationship between plasma biomarkers and SCI scores in Sleep+ group. SCI, Sleep Composite Index; Sleep +, high‐risk group; Sleep−, low‐risk group.

In the Sleep+ and Sleep− groups, the Sleep+ group showed significantly higher levels of plasma NfL compared to the Sleep− group (*p* < 0.05). However, no significant differences were observed in other plasma biomarkers (Figure 2A–D). Subsequently, we conducted correlation comparisons and found that in the Sleep+ group, Aβ42/40 was significantly correlated with SCI, and higher SCI scores were associated with lower Aβ42/40 levels, indicating that poorer overall sleep quality was associated with a higher risk of AD. No significant correlations were found for other plasma biomarkers (Figure 2E–H). Additionally, no other significant changes were observed in the overall sample and Sleep− group.

### 3.4 The interaction of SCI and APOE ε4 in predicting plasma biomarkers and cognitive decline

**FIGURE 3:**
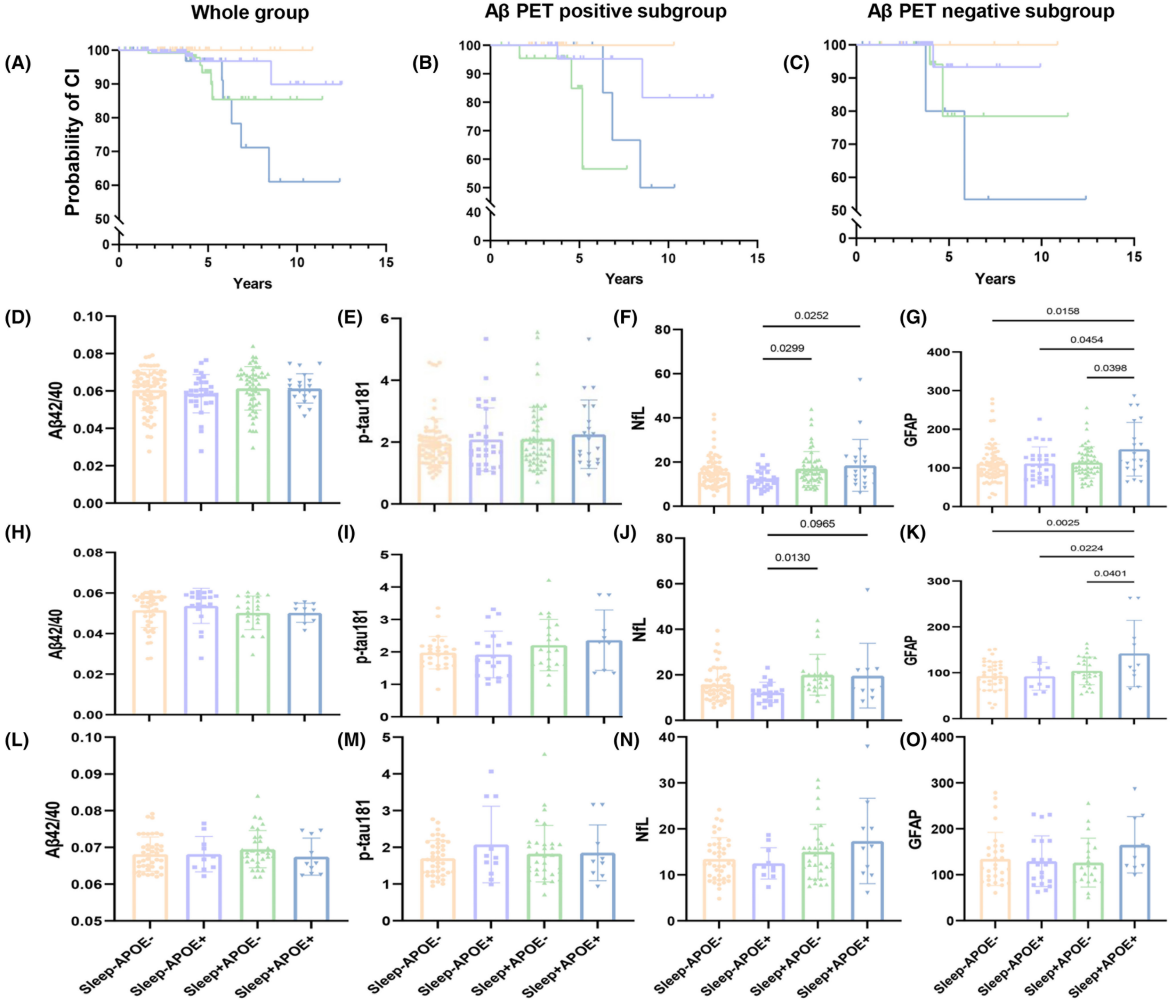
SCI and APOE ε4 genotype on future dementia risk and plasma biomarkers level. (A–C) Estimation of SCI and APOE ε4 genotype on future dementia risk. (A) Whole group, (B) Aβ PET‐positive subgroup, (C) Aβ PET‐negative subgroup; (D–O) SCI and APOE ε4 genotype on plasma biomarkers level; (D–G) whole group; (H–K) Aβ PET‐positive subgroup; (L–O) Aβ PET‐negative subgroup.

Figure 3A–C show the probabilities of dementia in the overall sample, Aβ PET‐positive group, and Aβ PET‐negative group. In all three groups, individuals with both Sleep+ and APOE+ have significantly higher dementia risk compared to the control group (Sleep−/APOE−), and individuals with Sleep+/APOE+ also have higher risk than those with Sleep−/APOE+. In the Sleep+ group, we observed that regardless of APOE risk gene carriage, both Sleep+/APOE+ and Sleep+/APOE− individuals exhibited poorer outcomes, significantly higher than the Sleep−/APOE− group.

In the overall sample (Figure 3D–G), the NfL levels in the Sleep−/APOE+ group were significantly lower than those in the Sleep+/APOE+ (*p* = 0.025) and Sleep+/APOE− (*p* = 0.030) groups. The GFAP levels in the Sleep+/APOE+ group were significantly higher than those in the Sleep+/APOE− (*p* = 0.040), Sleep−/APOE+ (*p* = 0.045), and Sleep−/APOE− (*p* = 0.016) groups. In the Aβ PET‐positive group (Figure 3H–K), similar differences were observed in NfL and GFAP levels. The NfL levels in the Sleep−/APOE+ group were significantly lower than those in the Sleep+/APOE− (*p* = 0.013) group, although the difference between Sleep+/APOE+ and Sleep−/APOE+ groups was marginal (*p* = 0.097). As for GFAP levels, the results were consistent with those in the overall sample, with the Sleep+/APOE+ group showing significantly higher levels compared to the Sleep+/APOE− (*p* = 0.040), Sleep−/APOE+ (*p* = 0.022), and Sleep−/APOE− (*p* = 0.003) groups. In the Aβ PET‐negative group, although similar trends were observed, no significant differences were found (Figure 3L–O).

### 3.5 Mediation analysis

**FIGURE 4:**
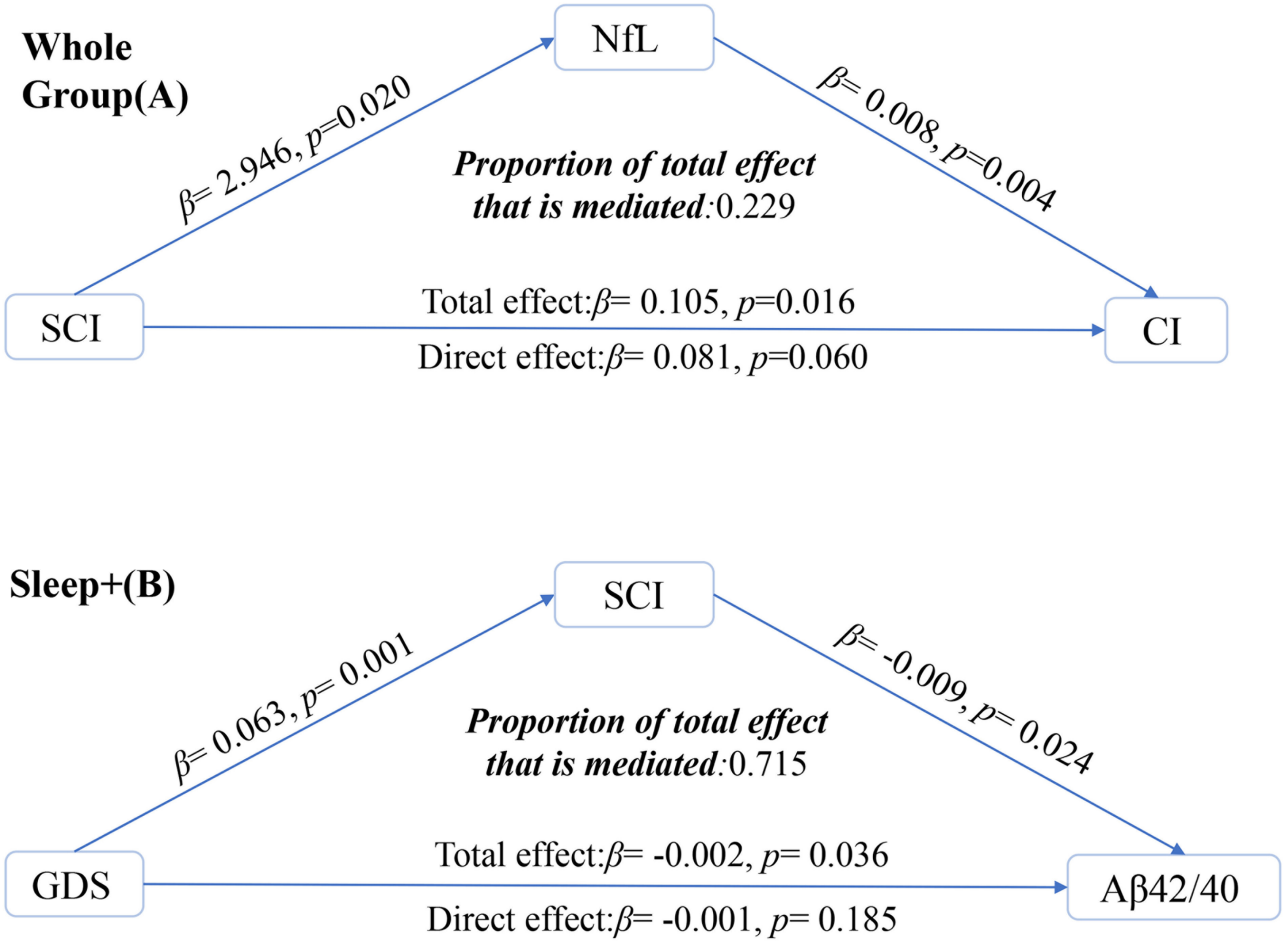
Mediation path diagram showing plasma NfL as a potential mediator between SCI (independent variable) and CI dependent variable (A). Mediation path diagram showing SCI as a potential mediator between GDS (independent variable) and plasma Aβ42/40 (dependent variable, B). CI, cognitive impairment; GDS, Geriatric Depression Scale; NfL, neurofilament light chain; SCI, Sleep Composite Index.

In our additional mediation analysis, we found that SCI had a significant indirect effect on CI through plasma NfL levels, which explained 22.9% of the total effect (Figure 4A). In the Sleep+ group, GDS scores had a significant negative indirect effect on plasma Aβ42/40 levels through SCI, which accounted for 71.5% of the total effect (Figure 4B). However, we did not observe any significant effects of GDS on other plasma biomarkers.

## 4 DISCUSSION

This large‐scale multimodal biomarker study utilized PSQI, RBDSQ, and ESS sleep‐related questionnaires to assess individuals' overall sleep status (represented by SCI) and examined the association of SCI, SCI‐APOE genotype subgroups, plasma biomarker levels related to AD, and longitudinal cognitive changes. We found that APOE ε4 as genetic risk factor could exacerbate effect of sleep disorder on risk conversion to dementia. These findings offer the potential for better identification of high‐risk individuals with normal cognition for early intervention and treatment, as well as a deeper exploration of the disease progression mechanisms in AD.

The current study supports that SCI as a composite measurement of sleep quality has good predictive power on the risk of dementia. According to the 2023 NIA‐AA Revised Clinical Criteria for AD, plasma biomarkers have gained recognition for their diagnostic and predictive value in AD.^28^ Consistent with some previous studies,^29^, ^30^, ^31^ our research confirmed the predictive role of plasma p‐tau181, NfL, and GFAP in future dementia risk. Our results suggested that each unit increase in p‐tau181 was associated with an 86.4% increased risk of dementia progression. Similarly, each unit increase in NfL and GFAP was associated with 8.4% and 1.8% increased risk of dementia progression, respectively. Importantly, our SCI showed a 4.37‐fold increased risk of dementia progression for each unit increase. The relationship between sleep and AD has been a topic of extensive discussion. A previous cohort study demonstrated that markers of poor sleep quality, such as decreased sleep efficiency and total sleep time (either too long or too short), were associated with cognitive decline.^13^ Sleep deprivation accelerates the release of Aβ and the formation of Aβ plaques, which, in turn, impair sleep quality and disrupt the sleep–wake cycle in brain regions controlling for sleep circadian.^7^ However, other sleep quality factors, such as sleep duration, disturbances, efficiency, and daytime dysfunction, were found to be unrelated to Aβ burden, indicating that plasma Aβ may not be an optimal predictor of prognosis.^29^ Sleep disturbances are even associated with cognitive impairment before the clinical onset of AD. A community‐based study showed a link between delayed sleep–wake cycles and reduced likelihood of developing dementia.^32^ Over a 6‐year follow‐up period, age‐related sleep fragmentation was associated with a 5.6‐fold increased risk of developing dementia.^33^ Additionally, a longitudinal cohort study revealed that excessive daytime sleepiness was associated with a twofold increased risk of dementia,^34^ indicating that daytime sleepiness contributes to dementia onset. Thus, previous studies focusing on specific aspects of sleep may not fully and comprehensively reflect the overall impact of sleep.

APOE ε4 is a genetic risk factor for developing AD.^35^, ^36^ Normal cognitive, elderly individuals carrying the APOE ε4 allele show a sevenfold higher risk of developing MCI or dementia.^37^ While some studies suggest that the ε4 allele may be the main cause of sleep disruption in elderly individuals at risk of dementia,^38^, ^39^ others emphasize that sleep deprivation and APOE genotype may only amplify each other's adverse effects.^40^ Therefore, we used a grouping approach that combines APOE ε4 allele carriers and non‐carriers (APOE +/APOE−) with high and low risk based on the SCI scores for analysis. The results showed that the dementia progression in the Sleep+/APOE+ group was faster than in the Sleep+/APOE− and Sleep−/APOE+ groups, and significantly faster than in the Sleep−/APOE− group. However, when using Aβ PET+ subgroups, dementia progression was accelerated considerably in the Sleep+ group regardless of APOE genotype (+/−). Hence, currently, the debate on the influence of ApoE‐ε4 remains open, as some studies have shown no influence^41^ or even a protective role of APOE genotype on sleep patterns, raising questions about the true nature of this association.^42^ Although further investigations are needed, there is a theoretical proposition that improved sleep consolidation can mitigate the increased risk associated with APOE genotype.^43^ Our results may also support this finding that improved sleep reduces the increased risk associated with the APOE genotype. Nevertheless, once individuals have reached the Aβ PET+ stage, the elevated risk of dementia is predominantly influenced by their sleep status rather than their APOE genotype.

Plasma biomarker concentrations appear to be influenced by underlying diseases.^44^ In our study, we observed that plasma NfL was significantly higher in the SCI+ group compared to the SCI‐ group. Within the SCI+ group, we also found a significant negative correlation between plasma Aβ42/40 and SCI, indicating that individuals with poorer overall sleep quality (higher SCI scores) had lower plasma Aβ42/40 levels. This finding is consistent with a previous cross‐sectional study, although they measured Aβ42/40 using CSF.^45^ Under the Sleep/APOE grouping system, we observed differences in NfL and GFAP levels among the groups. In the entire sample, NfL levels were significantly higher in the Sleep+/APOE+ group than in the Sleep+/APOE− group, and the latter had substantially higher NfL levels than the Sleep−/APOE+ group. This suggests that the effect of individual Sleep+ status on NfL levels may be more robust than that of APOE+ status, although direct evidence was not found. Furthermore, studies by Adriano et al.^10^ and Carvalho et al.^46^ have confirmed the potential role of NfL as a biomarker for disrupted sleep/excessive daytime sleepiness in patients with AD and its role in predicting neurodegeneration and cognitive decline. Higher NfL levels may be associated with sleep disruption and excessive daytime sleepiness through interference with connections or damage to arousal centers. It has been discovered that chronic sleep deprivation significantly increases GFAP levels, which are astrocytic‐specific markers.^11^ Similar findings were observed in our study; the GFAP level was higher in the Sleep+ group, especially the Sleep+/APOE+ ratio was significantly higher than in the other three groups. This suggests that when individuals are in a Sleep+ state and have the APOE+ genotype, the two factors may mutually influence and promote each other, leading to abnormally elevated GFAP levels. However, the specific mechanisms will require further in‐depth research in the future. The implications of our results extend beyond mere biomarker measurements. Elevated NfL and GFAP levels could potentially serve as indicators of disrupted sleep and sleep‐related disorders in individuals at risk for neurodegenerative diseases. This insight could guide early interventions and preventive strategies to mitigate cognitive decline and neurodegeneration. Furthermore, the relationship among sleep disturbances, APOE status, and astrocytic activation might offer novel avenues for therapeutic interventions targeting both sleep disorders and neurodegenerative conditions.

In our study, we also found a mediating effect of NfL levels between SCI and dementia. The impact of SCI on future dementia is partially mediated by NfL (proportion of total effect that is mediated: 0.229). The relationships between SCI and NfL, as well as NfL and dementia, have been confirmed in several previous studies.^10^, ^45^, ^46^ Interestingly, in the Sleep+ group, we observed that SCI fully mediated the relationship between GDS and Aβ42/40 (proportion of total effect that is mediated: 0.715). This finding is not surprising as previous research has reported a strong negative correlation between GDS and Aβ42/40.^47^ Existing studies demonstrated that GDS contributes to sleep disorder, and in return, sleep disorder exacerbates depression magnitude.^48^, ^49^ Moreover, long‐lasting depression state is significantly associated with left hippocampus volume, indicating that onset of first‐time depression is important in neurodegeneration in hippocampus that results in cognitive decline.^50^ The smaller the hippocampus volume, the worse the clinical prognosis, and more depression manifestation as well.^51^ Besides, accumulating evidence have stressed the role of generalized inflammation which is commonly seen in both cognitive decline and depression.^52^ Hence, the relationship between sleep disorder and cognitive decline is complicated and bidirectional. The mediation analyses provide valuable insights into the underlying mechanisms by which sleep quality, plasma biomarkers, and genetic factors interact to influence the risk of developing dementia.

Limitations of the current study were acknowledged. First, the Aβ PET imaging and sleep quality assessments were conducted on different dates, and only a subset of individuals underwent both plasma biomarker analysis and PET scans. This temporal mismatch may introduce potential confounding factors, especially considering that Aβ deposition is a relatively slow process that takes years to occur.^53^ Additionally, sleep habits are often chronic, particularly in the age group under study. Utilizing PSQI, RBDSQ, and ESS multidimensional sleep questionnaires allowed for effective sleep assessment. However, the association between these questionnaires and objective sleep measurements remains somewhat ambiguous.^54^ Therefore, ideal future studies should incorporate the use of actigraphy and/or polysomnography for objective sleep measurements in large‐scale cohorts. Lastly, the current study did not take into account the effect of sleep‐related medication on cognitive performance. Furthermore, expanding the sample size in future research would be beneficial in gaining a deeper understanding of the complex nature of these associations.

## 5 CONCLUSION

In conclusion, this study revealed the association between SCI and AD plasma biomarkers, as well as explored whether these associations were modulated by APOE ε4 allele carriage. In this cohort study of cognitively healthy elderly individuals, we found that sleep disruption is linked to future dementia, particularly in APOE ε4 carriers, which was partially explained by the increased plasma NfL concentrations. Additionally, we found that APOE ε4 carriers with sleep disorder had the highest plasma NfL and GFAP compared to the other groups. These findings are important for understanding the relationship between sleep disorder and AD progression, which may provide novel insights toward using sleep index and APOE ε4 genotyping to identify individuals with high risk of AD or cognitive decline in elderly adults.

## FUNDING INFORMATION

This study was funded by the National Natural Science Foundation of China (Grant No. 61633018, 82020108013, and 82171197) and the Guangdong Basic and Applied Basic Science Foundation for Distinguished Young Scholars (Grant No. 2023B1515020113).

## CONFLICT OF INTEREST STATEMENT

The authors declare that they have no competing interests.

## CONSENT TO PARTICIPATE

All participants provided written informed consent and authorized the publication of their clinical details. SILCODE is listed on the ClinicalTrials.gov registry (SILCODE: NCT03370744).

## CONSENT FOR PUBLICATION

The authors take full responsibility for the data, the analyses and interpretation, and the conduct of the research. The authors had full access to all of the data and had the right to publish any and all data separately and apart from any sponsor.

## Data Availability

The data used to support the findings of this study are available from the corresponding author upon request.
